# Possible factors affecting thyroid dysfunction in hepatitis C virus-infected untreated patients

**DOI:** 10.3892/etm.2014.1709

**Published:** 2014-05-14

**Authors:** XIAO-RONG MAO, LI-TING ZHANG, HONG CHEN, PING XIAO, YOU-CHENG ZHANG

**Affiliations:** 1 Department of Infectious Diseases, The First Hospital of Lanzhou University, Lanzhou, Gansu 730000, P.R. China; 2 Department of General Surgery, The Second Hospital of Lanzhou University, Lanzhou, Gansu 730030, P.R. China

**Keywords:** thyroid dysfunction, liver dysfunction, hepatitis type C, Chinese population

## Abstract

The present study investigated the association of thyroid dysfunction (TD) with the distribution of chronic hepatitis C virus (HCV) infection in untreated patients. A total of 1,012 cases of HCV-infected patients were collected from different regions, of which 209 patients demonstrated a type of TD (chronic thyroiditis complicated with hyperthyroidism, chronic thyroiditis complicated with hypothyroidism, subclinical hyperthyroidism, subclinical hypothyroidism, hyperthyroidism, hypothyroidism or chronic thyroiditis). The results showed the existence of geographical differences in the types of TD present with HCV infection. The female patients had a higher incidence of autoimmune-related TD than the male patients. High levels of HCV RNA expression were most common in all HCV-infected patients, regardless of the presence of TD. High and medium expression levels of HCV RNA were more prevalent in the patients with autoimmune-related TD. Relative analysis of the HCV RNA levels showed that the pathogenesis of TD was not correlated with the HCV RNA expression levels; however, it may have been associated with autoimmunity. The HCV-infected patients with TD were most commonly middle-aged, whereas young adults were the largest group of patients with HCV and normal thyroid function. Among all HCV genotypes, type 1b was the most common HCV genotype and type 2 was the second most common. Types 3 and 6 were scarce in this study population. No associations were identified between HCV genotypes and thyroid disease. The data of liver function showed that HCV-infected patients with TD had a higher liver dysfunction rate compared with that of the patients with normal thyroid function. Therefore, liver dysfunction may be associated with thyroid disease. This study supports the potential of individualized treatment for HCV-infected patients.

## Introduction

Hepatitis C (HCV) is the principal cause of chronic liver disease, cirrhosis and hepatocellular carcinoma (HCC). It was estimated in 2001 that the number of new cases of HCV infection worldwide is >3.5 million per year (1). In China, a country with a high incidence of HCV infection, there are ~40 million individuals infected with HCV, with a prevalence of ~3.2% (2). HCV infection has been verified to be associated with numerous autoimmune disorders, including thyroid dysfunction (TD), autoantibody formation and autoimmune idiopathic thrombocytopenic purpura (3,4). TD is one of the complex diseases common in patients with chronic HCV infection and is triggered by interactions between genetic, epigenetic and microenvironmental factors (5). Therefore, the association between the epidemiological factors of HCV infection and possible properties of TD requires investigation in patients with HCV infection. Although the high incidence of TD in patients with HCV receiving interferon-α therapy has been well verified in a previous study (6), the correlation between TD and HCV-infected patients without interferon treatment remains under debate and is explored in the present study.

TD may be divided into chronic thyroiditis complicated with hyperthyroidism, chronic thyroiditis complicated with hypothyroidism, subclinical hyperthyroidism, subclinical hypothyroidism, hyperthyroidism, hypothyroidism and chronic thyroiditis for clinical diagnosis. An increased incidence of clinical and subclinical autoimmune thyroiditis has been observed in patients with chronic HCV infection compared with that in healthy controls (7). Hypothyroidism is more common in patients with chronic HCV infection than in normal controls or patients with chronic hepatitis B infection (8). However, the reports on the correlation between chronic HCV infection and TD are limited, and are mainly focused on HCV patients during interferon treatment (9) or overall TD incidence (10). To the best of our knowledge, o study has investigated on the correlation between chronic HCV infection and each type of TD. TD has been reported to have an occurrence of 2–13% in patients with HCV infection, and is more frequent in females (11,12). The prevalence of TD in HCV-infected patients has been frequently reported; however, the correlation of multiple factors associated with TD, including gender, age, geographical location, HCV genotype and RNA expression levels, with HCV infection remains unknown, particularly in the Chinese population. The present study aimed to investigate the correlation between various types of TD and the epidemiological factors of HCV-infected patients, including geographical distribution, HCV RNA expression levels, HCV genotype, gender, age and liver function in different regions of China.

## Patients and methods

### Patient population

**Figure 1:**
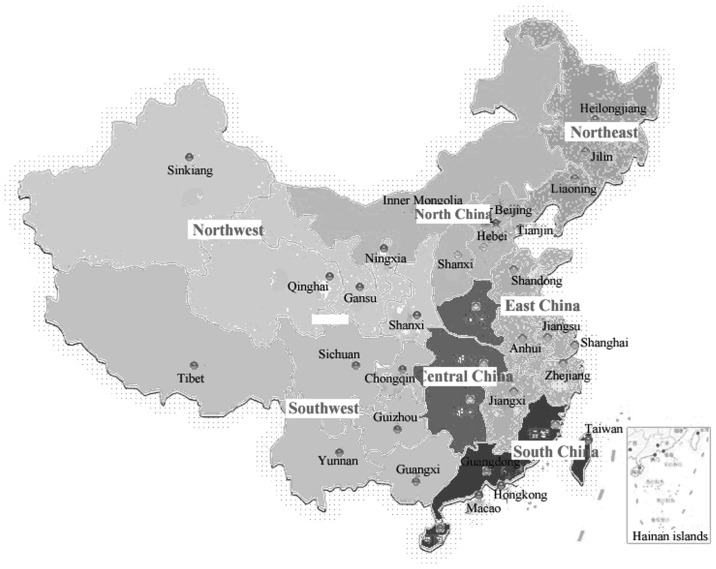
Regional distribution of the patients with HCV recruited in this study. Twenty-eight hospitals were divided territorially into North, Northeast, Southwest, South, Central, Northwest and East China. There were 92 HCV-infected patients in North China, 91 in Southwest China, 104 in South China, 224 in Central China, 162 in Northwest China, 252 in East China and 89 in Northeast China. HCV, hepatitis C virus.

The registration ID for the present study is the ClinicalTrials.gov identifier NCT01293279. A total of 1,012 patients infected with HCV, including 209 patients with a type of TD, were randomly recruited for this study. The patients were treated at 28 hospitals in different regions of China (Table I) and the age, gender and HCV RNA expression were considered. All the patients were free of cirrhosis and HCC, and had not been treated with interferon. The study was approved by the ethics committees of all hospitals at which the patients were recruited. Written informed consent was obtained from each patient. The hospitals were divided territorially into North, Northeast, Southwest, South, Central, Northwest and East China (Fig. 1). Among the 1,012 patients with HCV infection, there were 92 cases in North China, 91 in Southwest China, 104 in South China, 224 in Central China, 162 in Northwest China, 252 in East China and 89 in Northeast China.

### HCV genotypes and RNA expression levels

The specific primers and typing probes for the HCV genotyping were designed according to the 5′ non-coding region (nt299-1) of HCV gene sequences published in GenBank. The HCV RNA levels in the plasma samples of the patients with HCV genotypes 1b, 2, 3, 6 were detected by an Abbott RealTime HCV assay (Abbott Laboratories, Des Plaines, IL, USA), and total RNA was isolated from the plasma of the HCV-infected patients using an RNeasy Mini kit and a RNase-Free DNase set (Qiagen, Hilden, Germany). The PCR products were dot-blot hybridized to determine the HCV genotypes [Versant HCV Genotype 2.0 assay (LiPA); Siemens Healthcare Diagnostics, Tarrytown, NY, USA].

### Thyroid function

Peripheral blood samples were collected from fasting patients and were stored at −70°C until use. In each patient, the plasma levels of thyroid-stimulating hormone (TSH), thyroxine (T4), triiodothyronine (T3), free thyroxine (FT4), free triiodothyronine (FT3), and anti-thyroid peroxidase (anti-TPO) antibodies (Ab) were measured by the immune-chemiluminescense-assay method (ADVIA Centaur^®^ CP immunoassay System; Siemens AG, Erlangen, Germany). Anti-thyroglobulin Ab (anti-Tg Ab) was determined by the Immulite^®^ 1,000 Systems method (Siemens AG). Thyroid function was defined according to the criteria taken from Surks *et al* (13). Therefore, the subjects were divided into chronic thyroiditis complicated with hyperthyroidism, chronic thyroiditis complicated with hypothyroidism, subclinical hyperthyroidism, subclinical hypothyroidism, hyperthyroidism, hypothyroidism and chronic thyroiditis with normal thyroid function (Table II). Hyperthyroidism was defined with TSH levels <0.1 mIU/l and elevated FT4 level. Subclinical hyperthyroidism was defined as TSH levels between 0.1 to 0.4 mIU/l and normal FT4 levels. Hypothyroidism was defined as a TSH level ≥10 mIU/l independently of the FT4 value. Subclinical hypothyroidism was defined as a TSH level of 4.5 to 10 mIU/l, with an FT4 level between 10 to 25 pmol/l. Positive anti-TPO antibodies (>35 IU/ml) and anti-Tg antibodies (>40 IU/ml) were indications for complicated chronic thyroiditis.

### Liver function

The liver function was tested in each HCV patient. The concentrations of alanine aminotransferase (ALT), aspartate aminotransferase (AST) and albumin (ALB) were determined by automated clolrimetric method on Beckman SYNCHRON LX20 system (Beckman-Coulter, Fullerton, CA, USA). Reference ranges were: ALT 5–40 U/l, AST 8–40 U/l, ALB 35~55 g/l.

### Statistical analysis

Student’s t-test, χ^2^ test and Fisher’s exact test were performed to compare the differences among the groups of patients. Two-tailed P<0.05 was considered to indicate a statistically significant difference. The statistical analyses were performed with the statistical package SPSS, version 13.0 (SPSS, Inc., Chicago, IL, USA).

## Results

In this study, all 1,012 HCV-infected patients treated at 28 hospitals in 7 regions of China were randomly recruited (Fig. 1; Table I). The gender ratio of males to females was 552/460. The HCV-infected patients were divided into four groups according to age: The elderly (≥60 years old), middle-aged (45–59 years old), young adults (18–44 years old), and teenagers (<18 years old). The HCV genotypes 1b, 2, 3 and 6 were detected in the recruited patients. Furthermore, the HCV RNA expression levels were detected and divided into high (RNA≥1×10^7^ copies/ml), medium (1×10^5^≤RNA<1×10^7^ copies/ml) and low (RNA<1×10^5^ copies/ml).

### Incidence of TD in patients with HCV infection in different regions

The distribution of various types of TD in HCV-infected patients was investigated. The study showed that North (28.3%) and Northwest (26.5%) China accounted for the highest proportions of TD in patients with HCV infection, and the lowest proportions were observed in the patients in Southwest (15.7%), South (14.4%) and Central (17.4%) China. Of the various types of TD, hyperthyroidism had the lowest incidence in the TD patients with HCV infection. Subclinical hypothyroidism had the highest incidence of all the types of TD; the highest proportions of patients with subclinical hypothyroidism were in North (13.0%) and North West (14.2%) China and the lowest proportion was in South China (1.9%). Chronic thyroiditis with normal thyroid function was observed at the highest proportions in North East (10.1%) and North (8.7%) China (Table III).

### Distribution of the HCV genotypes and RNA expression levels in TD patients with HCV infection

Numerous studies have verified the association between autoimmune disorders and HCV infection (14,15). Therefore, the HCV genotypes and RNA expression levels in patients with autoimmune-related TD and other types of thyroid disease were detected in the present study. The data showed that the patients with medium expression levels of HCV RNA were the predominant population of those with chronic thyroiditis complicated with hypothyroidism (54%, 15/28). Patients with high and low HCV RNA expression levels were the main population of those with hypothyroidism, with percentages of 56 (10/18) and 38% (7/18), respectively. The proportion of patients with HCV and high and medium HCV RNA expression levels was higher in those with autoimmune-related TD than in the patients with normal thyroid function (Table IV). HCV genotyping shows that among all genotypes, 1b was the most common genotype in HCV-infected patients with TD and without TD, and the percentages were 61% and 58%, respectively (Table V).

### Distribution of TD in patients with HCV infection of different genders and ages

The data showed that in all recruits, the numbers of the elderly, middle-aged, young adults and teenagers accounted for 14.8 (150/1,012), 37.7 (382/1,012) and 47.3 (480/1,012) (the two teenagers were excluded from the TD analysis), respectively. In all HCV-infected patients with TD, the middle-aged group (43.5%) accounted for the largest number, which increased to 72.2% in the HCV-infected patients with hypothyroidism. However, young adults were the main group (52.2%) of HCV-infected patients without TD. Gender differences were identified in the numbers of HCV-infected patients with TD. The percentage of female patients was significantly higher than that of the males in the total number of patients with TD. However, the percentage of males was higher than that of females in the number of HCV-infected patients without TD (Table VI).

### Association of liver function with TD in HCV-infected patients

The prevalence of liver disorders in patients with TD and HCV infection was also investigated in the present study. The data showed that the incidence of a liver disorder in the patients with chronic thyroiditis complicated with hypothyroidism (46.5%) was lower than that in patients with other types of TD. The patients with hypothyroidism had the highest percentage of liver disorder (69%) among all HCV-infected patients. The results for hyperthyroidism are of no statistical significance due to the small sample size. Although lower ALB levels were common in HCV-infected patients with normal thyroid function, patients with low levels of ALB were scarce in those with any type of TD (Table VII).

## Discussion

HCV infection is a major cause of chronic liver disease worldwide. Varied data on the prevalence of HCV infection are frequently reported in studies (16,17). These discrepancies reflect not only the distinct epidemiological characteristics of patients with HCV, but also the differences in the methodologies used. Taking into account the impact of external and anthropic factors, all samples were analyzed in the same center in the present study.

A previous study has demonstrated that the distribution of the HCV genotypes demonstrates geographical differences, and this impact should be considered in an epidemiological study (18). The HCV genotypes are considered a major determinant of the response to treatment in HCV infection (19,20). Therefore, the status of HCV infection and the HCV genotype are important in the etiological diagnosis, clinical treatment and vaccine development of HCV infection in specific regions. However, no significant difference in the distribution of HCV genotypes was identified between the TD and normal thyroid function groups in the present study. This indicates that the geographical differences in the TD incidence in patients with HCV infection is not likely to be caused by the HCV genotype.

Certain studies have shown that hypothyroidism and thyroid autoimmunity disorder are more common in patients with HCV, even in the absence of interferon treatment (5,21). However, the present study shows that subclinical hypothyroidism is the most prevalent type of TD in untreated HCV patients, while patients with hyperthyroidism account for the lowest proportion of those with HCV infection. Furthermore, the HCV RNA expression levels in patients with different types of TD were detected. The data confirm the existence of an association between TD and the HCV RNA expression levels. Patients with medium expression levels of HCV RNA are the predominant population of those with chronic thyroiditis complicated with hypothyroidism (53.7%, 15/28), but are scarce in those with hypothyroidism. Low levels of HCV RNA are common in patients with hypothyroidism. This result indicates that the pathogenesis of TD is not likely to be associated with the HCV RNA expression levels but may be associated with autoimmunity. In HCV-infected patients, autoimmunity is an important pathogenesis of TD development through the actions of thyroid autoantibodies and certain chemokines (22,23) The detailed mechanisms require investigation in the future.

Previous studies have shown that gender is one of the most common risk factors that predict the development of TD during interferon therapy (7,24,25). TD is more common in females than in males in different regions all over the world, which has been verified in the majority of conditions caused by HCV infection and other diseases (26,27). In the present study, it was also identified that the number of females was significantly larger than that of the males in the cohort of patients with TD and HCV infection. However, in China HCV infection is more prevalent in males than in females (28), leading to markedly more male patients than female patients. Hsieh *et al* reported that the female gender is a predisposing factor for TD in patients with HCV infection receiving interferon treatment (29), which is supported by the findings of the present study in Chinese patients. The incidence of TD in patients with HCV of different age groups was also investigated. The results show that patients with TD are mainly distributed in the middle-aged group. Furthermore, the percentage of patients with TD among middle-aged patients with HCV infection is highest in those with hypothyroidism (72.2%). Cappola *et al* reported the percentage of each type of TD in 3,233 normal elderly adults. The study demonstrated that subclinical hypothyroidism was the most prevalent type and accounted for 15.3% of all recruited cases (30), which is in accordance with the results of the present study.

The incidence of liver disorders in TD patients was investigated and it was identified that the percentage of patients with liver disorder was highest in those with hypothyroidism (69%) than in other types of TD (P<0.05). A correlation between hypothyroidism and liver disorders may exist in HCV-infected patients. However, as patients with chronic thyroiditis complicated with hypothyroidism show a lower percentage of liver disorders than those with hypothyroidism, the higher incidence of liver disorder in patients with hypothyroidism may be unrelated to autoimmune disorder.

In conclusion, this study investigated the correlation between the TD subtypes and the geographical distribution, HCV RNA expression levels, HCV genotype, gender, age and liver function in HCV-infected patients of China. It was demonstrated that the highest incidence of TD in HCV-infected patients was in those from North and Northwest China. The HCV-infected patients with TD exhibited a higher frequency of high and medium RNA levels compared with that in the patients with normal thyroid function. Middle-aged patients account for the largest number of all HCV-infected patients with TD, while young adults are the main group of HCV-infected patients without TD. Hypothyroidism in patients with HCV is associated with a higher incidence of liver disorders. This study indicates differences in age, gender and geographical distribution among various types of TD in HCV-infected patients, which may aid the success of individualized treatment for HCV-infected patients.

## Figures and Tables

**Table I:** Regional information of the Chinese patients with HCV infection enrolled in this study.

Region	Hospital	No. of patients
North	Peking University People’s Hospital	59
	Beijing Friendship Hospital	33
Northeast	Shengjing Hospital of China Medical Hospital	34
	The First Hospital of Jilin University	34
	The Second Affiliated Hospital of Harbin Medical University	21
Southwest	West China Hospital, Sichuan	22
	The First Affiliated Hospital of Kunming Medical College	40
	Southwest Hospital	29
South	The First Affiliated Hospital of Guangxi Medical University	40
	Nanfang Hospital	14
	The Third Affiliated Hospital of Sun Yat-sen University	50
Central	Henan Provincial People’s Hospital	95
	The First Affiliated Hospital of Zhengzhou University	28
	People’s Hospital of Hubei Wuhan University	38
	Affiliated Tongji Hospital of Tongj Medical College of Huazhong University of Science and Technology	32
	The Second Xiangya Hospital of Central South University	31
Northwest	Tangdu Hospital	40
	The First Affiliated Hospital of Shanxi Medical University	37
	The First Affiliated Hospital of Lanzhou University	58
	Ningxia People’s Hospital	27
East	The First Affiliated Hospital of Nanchang University	37
	The First Affiliated Hospital of Anhui Medical University	19
	The Second Hospital of Shangdong University	41
	The First Affiliated Hospital of Medical College Zhejiang University	33
	The First Affiliated Hospital of Fujian Medical University	11
	Shanghai Ruijin Hospital	52
	The First Affiliated People’s Hospital of Shanghai Jiaotong University	2
	Jiangsu Province Hospital	57

HCV, hepatitis C virus.

**Table II:** Criteria of the different types of TD.

Types of TD	Tg	Tb	TSH	T3	T4	FT3	FT4
Chronic thyroiditis complicated with hypothyroidism	+	+	↑	↓/not	↓	↓/not	↓
Subclinical hypothyroidism	−	−	↑	NOR	NOR	NOR	NOR
Chronic thyroiditis complicated with hyperthyroidism	+	+	↓	↑	↑	↑	↑
Subclinical hyperthyroidism	−	−	↓	NOR	NOR	NOR	NOR
Chronic thyroiditis with normal thyroid function	+	+	NOR	NOR	NOR	NOR	NOR
Hypothyroidism	−	−	↑	↓	↓	↓/not	↓/not
Hyperthyroidism	−	−	↓	↑	↑	↑	↑

TD, thyroid dysfunction; Tg, anti-thyroglobulin; Tb, anti-thyroid peroxidase; TSH, thyroid-stimulating hormone; T3, triiodothyronine; T4, thyroxine; F, free; +, positive; -, negative; ↑, elevated; ↓, reduced; NOR, normal. Hyperthyroidism was defined with TSH levels <0.1 mIU/L and elevated FT4 level. Subclinical hyperthyroidism was defined as TSH levels between 0.1 to 0.4 mIU/l and normal FT4 levels. Hypothyroidism was defined as a TSH level ≥10 mIU/l independently of the FT4 value. Subclinical hypothyroidism was defined as a TSH level of 4.5 to 10 mIU/l, with an FT4 level between 10 to 25 pmol/l. Positive anti-TPO antibodies (>35 IU/ml) and anti-Tg antibodies (>40 IU/ml) were indications for complicated chronic thyroiditis.

**Table III:** Geographical distribution of each type of TD in patients with HCV infection from different regions of China (%).

Types of TD	North	Northeast	Southwest	South	Central	Northwest	East
Chronic thyroiditis complicated with hyperthyroidism	0	0	0	3.8	0.9	1.2	1.2
Hyperthyroidism	1.1	0	0	0	0	0	0.4
Subclinical hyperthyroidism	0	0	0	1.0	0	0.6	0.8
Chronic thyroiditis complicated with hypothyroidism	1.1	4.5	3.4	1.0	2.8	2.5	3.6
Hypothyroidism	4.3	0	1.1	0	1.8	3.1	1.6
Subclinical hypothyroidism	13.0	7.9	6.7	1.9	8.0	14.2	7.1
Chronic thyroiditis with normal thyroid function	8.7	10.1	4.5	6.7	4.0	4.9	6.0
Total	28.2	22.5	15.7	14.4	17.5	26.5	20.7

TD, thyroid dysfunction; HCV, hepatitis C virus.

**Table IV:** HCV RNA expression levels in HCV-infected patients with different types of TD.

	RNA expression levels (%)		
	
Types of TD	High	Medium	Low
Chronic thyroiditis complicated with hyperthyroidism	55 (6/11)	45 (5/11)	0
Hyperthyroidism	100 (2/2)	0	0
Subclinical hyperthyroidism	50 (2/4)	50 (2/4)	0
Chronic thyroiditis complicated with hypothyroidism	32 (9/28)^a^	54 (15/28)	14 (4/28)
Hypothyroidism	56 (10/18)	6 (1/18)^a^	38 (7/18)^a^
Subclinical hypothyroidism	53 (46/86)	38 (33/86)	9 (7/86)
Chronic thyroiditis with normal thyroid function	48 (29/60)	42 (25/60)	10 (6/60)
Normal thyroid function	53 (430/803)	41 (326/803)	6 (49/803)

a Significant differences from the normal thyroid function with HCV infection group.

TD, thyroid dysfunction; HCV, hepatitis C virus.

**Table V:** Distribution of HCV genotypes in patients with TD and HCV infection.

	HCV genotype (%)			
	
Types of TD	1b	2	3	6
Chronic thyroiditis complicated with hyperthyroidism	64 (7/11)	18 (2/11)	9 (1/11)	9 (1/11)
Hyperthyroidism	100 (2/2)	0	0	0
Subclinical hyperthyroidism	50 (2/4)	0	25 (1/4)	25 (1/4)
Chronic thyroiditis complicated with hypothyroidism	61 (17/28)	32 (9/28)	3.5 (1/28)	3.5 (1/28)
Hypothyroidism	50 (9/18)	33 (6/18)	17 (3/18)	0
Subclinical hypothyroidism	60 (52/86)	35 (30/86)	2.3 (2/86)	2.3 (2/86)
Chronic thyroiditis with normal thyroid function	67 (40/60)	23 (14/60)	6.7 (4/60)	3.3 (2/60)
Normal thyroid function	58 (473/803)	24 (190/803)	10.3 (83/803)	7.7 (57/803)

HCV, hepatitis C virus; TD, thyroid dysfunction.

**Table VI:** Distribution (%) of the types of TD in patients with HCV infection in different gender and age groups.

Types of TD	Male	Female	Elderly	Middle-aged	Young adult
Chronic thyroiditis complicated with hyperthyroidism	45 (5/11)	55 (6/11)	27.3 (3/11)	36.4 (4/11)	36.4 (4/11)
Hyperthyroidism	0	100 (2/2)	50 (1/2)	0	50 (1/2)
Subclinical hyperthyroidism	75 (3/4)	25 (1/4)	25 (1/4)	25 (1/4)	50 (2/4)
Chronic thyroiditis complicated with hypothyroidism	29 (8/28)^a^	71 (20/28)	28.6 (8/28)^a^	50.0 (14/28)^a^	24.1 (6/28)^a^
Hypothyroidism	28 (5/18)^a^	72 (13/18)	5.6 (1/18)	72.2 (13/18)^a^	22.2 (4/18)^a^
Subclinical hypothyroidism	48 (41/86)	52 (45/86)	27.9 (24/86)^a^	39.5 (34/86)	31.4 (27/86)^a^
Chronic thyroiditis with normal thyroid function	40 (24/60)^a^	60 (36/60)	25 (15/60)^a^	43.3 (26/60)	31.7 (19/60)^a^
Normal thyroid function	58 (466/803)	42 (337/803)	12.1 (97/803)	35.1 (290/803)	51.8 (416/803)

a Significant differences from the normal thyroid function group.

TD, thyroid dysfunction; HCV, hepatitis C virus.

**Table VII:** Liver function with TD in HCV infected patients.

	Liver function		
	
Types of TD	ALT↑	AST↑	ALB↓
Chronic thyroiditis complicated with hyperthyroidism	7/11	5/11	0
Hyperthyroidism	0	1/2	1/2
Subclinical hyperthyroidism	1/4	3/4	1/4
Chronic thyroiditis complicated with hypothyroidism	14/28	12/28	3/28
Hypothyroidism	13/18	12/18	1/18
Subclinical hypothyroidism	48/86	50/86	8/86
Chronic thyroiditis with normal thyroid function	35/60	36/60	7/60
Normal thyroid function	517/803	448/803	64/803

TD, thyroid dysfunction; HCV, hepatitis C virus; ALT, alanine aminotransferase; AST, aspartate aminotransferase; ALB, albumin; ↑, elevated; ↓, reduced.
